# Low-Level Social Demand Is Associated with Anxiety-Related Gamma Wave Responses in Autistic Male Youth

**DOI:** 10.3390/brainsci15010040

**Published:** 2025-01-02

**Authors:** Vicki Bitsika, Christopher F. Sharpley, Ian D. Evans, Christopher B. Watson, Rebecca J. Williams, Kirstan A. Vessey

**Affiliations:** Brain-Behaviour Research Group, School of Science & Technology, University of New England, Armidale, NSW 2351, Australia; vicki.bitsika@une.edu.au (V.B.); ievans3@une.edu.au (I.D.E.); cwatso29@myune.edu.au (C.B.W.); rwilli90@une.edu.au (R.J.W.); kvessey@une.edu.au (K.A.V.)

**Keywords:** autism, social cognition, anxiety, gamma

## Abstract

Background: The Autism Spectrum Disorder (ASD) characteristic of difficulties in social communication and interaction has been previously associated with elevated anxiety and the degree of mental effort required to understand and respond to social cues. These associations have implications for the mental health of autistic youth, but they are usually based on correlational statistics between measures of anxiety and social interaction demands that are collected in formal psychological testing settings. Another index of mental effort that has been found to correlate with anxious arousal is gamma wave activity, which is measured via EEG. Methods: To compare data from both of these indicators of mental effort and anxiety, a two-stage study was conducted using (1) standardized test data and (2) in vivo EEG data in a low-demand social setting. Results: As well as significant associations between social cognition and anxiety from standardized scales, there were also meaningful relationships between social cognition and gamma wave activity. Conclusions: Because gamma wave activity represents the highest level of cognitive complexity for brain activity, is an index of hypervigilance under threatening conditions, and has been associated with anxiety in autistic youth, these findings suggest that even low-level demand social interaction settings may initiate high-level anxiety-related behaviour in autistic youth.

## 1. Introduction

### 1.1. Autism, Social Communication and Interaction

As well as exhibiting restricted and repetitive behaviours, people with Autism Spectrum Disorder (ASD) are characterized by difficulties in social communication and interaction [1]. Those social difficulties have been described by Constantino and Gruber [2] as responses to four stages or tasks: Social *Awareness* of social cues present in the environment; Social *Cognition*, or ability to understand what is being communicated to them; Social *Communication* (the actual social behaviour that is needed to engage socially); and Social *Motivation*, or how much the person values the opportunity to engage in social interaction. Difficulties in managing each of these four stages/tasks can result in impaired social interaction and communication problems that impede education and employment success [3].

### 1.2. Anxiety, Problem Solving in Social Cognition

In addition, there is evidence of a complex interaction between cognitive function, anxiety, and social cognition [4], particularly as it is focused towards the increased mental effort needed to understand social cues [5]. This association may be more complex than is immediately apparent with evidence of interactions between Constantino and Gruber’s [2] four stages of social communication and anxiety [6]. These findings suggest that there is some degree of anxiety in being able to bring sufficient cognitive effort to the issue of how to respond effectively in social situations, and that the autistic person’s mental health may be strongly influenced by these factors.

Some of those social cues faced by the autistic child may be perceived as threatening [7], initiating high-intensity neurocognitive responses aimed at understanding the nature of those social cues, and the appropriate social response to them (i.e., the processes underlying Social *Cognition*). The scale and complexity of these neurocognitive responses can be indicated via the detectable waves of electrical activity that move across the brain.

### 1.3. Brain Wave Activity and Cognitive Effort

Different types or intensities of cognitive effort may be indicated by the frequencies of brain activity that sweep across brain regions during various mental tasks. For example, during periods of daydreaming or relaxation not associated with fatigue, humans usually exhibit alpha activity (8–12 Hz). Faster frequencies include beta waves (13–30 Hz), which are associated with concentration (increases in beta wave activity have been associated with an increase in the difficulty of mental attention tasks which are also accompanied by increased concentration and mental stress [8]). Gamma waves (30–80 Hz) occur under conditions of maximum concentration and cognitive effort, such as the processing of highly complex information, working memory, and attentional processes [9], and they are reflective of the autistic individual’s lack of self-confidence in managing social interaction tasks [10].

Based on these findings, gamma band power may be considered as an index of high mental workload and cognitive arousal, followed by beta wave activity. When applied to the demands of understanding and responding to social interaction (i.e., Social *Cognition*), gamma and beta activity represents higher levels of cognitive effort than alpha activity particularly for persons who find that activity significantly challenging (such as autistic youth faced with social interaction challenges).

Thus, (1) the relative mental effort required to undertake the kinds of cognitive activities that underly each of the four stages of social communication and interaction that pose particular challenges for autistic people [1], i.e., Social *Awareness*, Social *Cognition*, Social *Communication*, and Social *Motivation*, might be indicated by the predominant frequencies of brain wave activity (i.e., alpha, beta, gamma) that are correlated with scores on those four stages. Furthermore, (2) because each of these four stages may require different cognitive skills or techniques that may be most likely to occur in specific parts of the brain, those brain regions in which the predominant frequency of brain wave activity occurs may provide additional information regarding the combination of *type* and *intensity* of that cognitive activity. Finally, (3) the presence of a background level of high cognitive activity (i.e., beta, gamma waves) might suggest that despite their external appearance, autistic youth remain in an elevated state of cognitive arousal (and possibly also physiological anxiety) even when they are not being faced with an obvious challenge to engage socially with others.

### 1.4. Aims of the Study

Therefore, to investigate these brain-based indices of the autistic persons’ response to the four-stage challenge of social communication and interaction, this study measured data from a two-step research protocol. First, there was a pre-laboratory data collection of participants’ Social *Awareness*, Social *Cognition*, Social *Communication*, and Social *Motivation* via a standardized scale completed by their parents about their child’s behaviour during the last six months plus the participants’ self-reported generalised anxiety state during the last two weeks. Second, there was a laboratory-based study in which participants’ alpha, beta, and gamma wave activity were collected during a condition of social unfamiliarity but relatively low social demand.

**Hypothesis 1.**  *There would be a significant correlation between participants’ Social Awareness, Social Cognition, Social Communication, and Social Motivation and their generalised anxiety score as an indicator of their ‘normal’ or ‘background’ anxiety state regarding the demands of these four stages of social communication and interaction.*

**Hypothesis 2.**  *During the low social demand laboratory condition, there would be a significant correlation between participants’ Social Awareness, Social Cognition, Social Communication, and Social Motivation and their beta and gamma wave activity but not their alpha activity.*

Support for each of these two hypotheses will assist in providing a clearer understanding of the underlying anxiety–cognitive arousal states of autistic youth with particular reference of the four stages of social communication and interaction described by Constantino and Gruber [2].

Because this study is one in a series focusing upon understanding how autistic youth manage their social communication and interaction challenges, and because most autistic persons are male, a sample of young autistic males was recruited.

## 2. Materials and Methods

### 2.1. Participants

This study is one of a series focused upon aspects of EEG data collected from a sample of male youth with ASD [11] in an endeavour to explore the associations between brain electrophysiological activity, anxiety, and diagnostic aspects of ASD. G*Power 3.1 power analysis showed that for a correlational study (i.e., the major statistical procedure used to test for associations between the SRS-2 subscales and EEG data), a sample size of 40 was sufficient to detect a medium effect [12] of *ρ* = 0.37, with α = 0.05 and power = 0.80. A sample of 41 participants was recruited from autism support groups on the Gold Coast, Australia (*M* age = 10.7 yr, SD = 3.1, range = 6 yr to 17 yr). Inclusion criteria were that the autistic boys were male by reference to their birth sex, aged between 6 and 18 years, had received a formal diagnosis of ASD from a psychiatrist or paediatrician, had an IQ ≥ 70 (to control for the effects of cognitive impairment), and had an ADOS-2 score of at least 7 or more. Boys were excluded if they had a history of epilepsy or schizophrenia or an intake of anticonvulsant medication. One parent of each child was also recruited as participants to accompany their son to the experimental laboratory and to give written consent to their son’s participation. Although not intentional, all these parents were their sons’ mothers. EEG data were collected at the Centre for Autism Spectrum Disorder laboratory at Bond University with ethics approval from the Bond University Human Research Ethics Committee (BUHREC Approval Number: 15786). EEG signal processing and data analyses were conducted in the Behavioral Neuroscience Laboratory at the University of New England (UNE Human Research Ethics Committee Approval Number: HE17-208).

### 2.2. Instruments

#### 2.2.1. The Autism Diagnostic Observation Schedule Second Edition (ADOS-2)

The ADOS-2 [13] is a highly recommended diagnostic observation tool for ASD [14,15], and it was used to confirm the previous clinician diagnosis of ASD by having an ADOS-2 Overall Total SA + RRB score of 7 or more as recommended by the ADOS-2 authors [13].

#### 2.2.2. The Wechsler Abbreviated Scale of Intelligence (2nd Edition) (WASI-II)

The WASI-II [16] is a screening test of intelligence that possesses strong validity with the WISC-IV, and it may be used instead of the WISC-IV with children. It contains four subtests with average reliability coefficients of between 0.92 and 0.96. The WASI-II provides a quick and accurate estimate of full-scale intelligence that is useful for research screening purposes.

#### 2.2.3. The Social Responsiveness Scale (2nd Ed.) (SRS-2)

The SRS-2 [2] measures the domains of interpersonal behaviour, communication, and repetitive/stereotypic behaviour based on the diagnostic criteria for ASD [1] in children between 4 yr and 18 yr of age. A total of 65 items produce scores on five subscales (Social *Awareness*, Social *Cognition*, Social *Communication*, Social *Motivation*, Restricted Interests and Repetitive Behaviour [RRB]). Caregivers answer the SRS-2 items about a familiar child “as they best describe your child’s behaviour over the last 6 months”, using responses ranging from 0 (not true), 1 (sometimes true), 2 (often true) and 3 (almost always true) so that high scores on these five subscales indicate greater difficulties in the relevant domains. The SRS-2 has a Cronbach’s alpha in excess of between 0.76 and 0.91. 90; similar data are available for the five subscales, which range from 0.76 to 0.91 (median = 0.85). To avoid a possible confound due to different numbers of items in the SRS-2 subscales, T scores were used in this study, and the RRB data were not included.

#### 2.2.4. The Child and Adolescent Symptom Inventory 4th Revision (CASI-4)

The CASI-4 [17] is a multi-subscale inventory based on DSM-5-TR diagnostic criteria for children and adolescents. It has been used and normed on samples of autistic children and adolescents. Test–retest reliability is 0.67 (*p* < 0.001) over a six-week period with internal consistency (Cronbach’s alpha) of 0.74 [17]. One of the CASI-4 subscales is for Generalised Anxiety Disorder (GAD), consisting of 8 items measuring the diagnostic criteria for GAD from the DSM-5-TR [1]. The CASI-4-GAD may be self-completed by autistic youth using a self-rating of 0 (never), 1 (sometimes), 2 (often), or 3 (very often) for how they have felt about the 8 GAD symptoms “during the last two weeks”, thus providing a measure of severity beyond that from categorical assessment procedures.

### 2.3. EEG

A 40-channel NuAmps EEG amplifier from *Compumedics NeuroScan, Compumedics Ltd.* Melbourne, Australia, three Quik-Caps, *Compumedics Ltd.* (varying in size—small, medium, large) with 34 sintered Ag/AgCl electrodes, four drop-down integrated electrodes, and two auricle electrodes was used to collect the EEG data. Cz was chosen as the reference electrode.

### 2.4. Procedure

#### 2.4.1. Step 1

Mothers and their autistic sons visited the first author’s laboratory to view the EEG equipment, have the experimental procedure described to them, and complete the SRS-2 (mothers) and CASI-4-GAD (boys).

#### 2.4.2. Step 2

During the second stage of the study, mothers and sons revisited the lab, gave written consent (mothers and boys aged 15 years or more) or assent (boys aged 6 years to 14 years), and underwent the experimental procedure described below in a sound-attenuated laboratory of approximately 4 m × 5 m.

#### 2.4.3. EEG

The EEG recording equipment was positioned behind the boys, each of whom sat on a sofa chair. A camera was used to record the boys’ responses, as well as any anxious behaviour and physiological responses that might interfere with EEG data collection. The experimental protocol was led by a research-capable assistant who had worked with autistic children for several years and who sat behind the autistic boy during the procedure.

As mentioned above (Section 2.1), the data reported here are part of a larger study [11], and only the relevant procedures are reported. Boys underwent an adaptation period for 15 min, during which the EEG cap was fitted, and they sat quietly and engaged in low-level conversation with the experimenter. Following this, the boys experienced three minutes of resting eyes closed conditions, following standardised recommendations [18,19], and these data were used in this study. During this 3-minute resting eyes closed condition, no other personnel were present in the setting, and no further social interaction with the participants was included. After the experiment was complete, the boys had the cap removed, their skull was cleaned where necessary, they were asked whether they had any questions, and they were thanked for their participation.

#### 2.4.4. Data Acquisition and Pre-Processing

The sampling rate was 1 kHz. Impedances were set at or below 5 kΩ. Due to sensory sensitivities that are characteristic in this group of participants, the experimenter was mindful to limit abrasion of the scalp.

#### 2.4.5. EEG Signal Processing

All EEG data collected were treated with a constant baseline correction to eliminate any DC offsets. Filter parameters included the notch filter with harmonics (frequency: 50 Hz; slope: 1.5 Hz) and the bandpass with both low (frequency: 0.5 Hz; slope: 2 Hz) and high (frequency: 30 Hz; slope: 5 Hz) filter settings. Data tapering was conducted with a Hann filter (width: 5%). Visual inspection identified any bad blocks, which were rejected. Automatic features offered in Curry 7 were applied to eye blinks, lateral or roving eye movements, electrode, and muscle sources to remove artifacts. Data were redefined into 2 s epochs, from which power spectra were derived via Fourier spectral analysis, plus Hann tapering applied with a width of 5%. The frequencies of interest were 8 to 12 Hz (alpha), 13.1 to 30 Hz (beta), and 30 to 80 Hz (Gamma).

### 2.5. Statistical Analysis

Data were analysed with the Statistical Package for Social Sciences (SPSS), version 27. Because of the elevated likelihood of a Type I error due to the large number of correlation coefficients being calculated, and the need to balance Type I and Type II errors within a limited sample size, the effect size was used as the determinant of a meaningful result rather than the *p* value, as recommended [20], and following Cohen’s [12] categorisation of ‘small’ = 0.10 to 0.29, ‘medium’ = 0.30 to 0.49, and ‘large’ = 0.5 to 1.0 effect sizes. Although the practice of normalising EEG data is common, there are strong theoretical and empirical arguments against it [21], and there is also some evidence that it can bias results [22]. A simpler solution is to apply statistical procedures that are capable of analysing non-normalised data, and this was accomplished via Spearman’s correlation in the current study. Sources of alpha, beta, and gamma band power were identified using *eLORETA* (exact low-resolution brain electromagnetic tomography) [23]. Based on the scalp-recorded electric potential distribution, *eLORETA* software 2002 computes the three-dimensional distribution of current source density (CSD) of 5 mm cubic voxels throughout the grey matter.

## 3. Results

### 3.1. Scale Data

Table 1 presents the means and standard deviations of the WASI-II Full Scale IQ data, ADOS-2 Overall Total SA and RRB scores, SRS-2 subscale T Scores, and CASI-4-GAD scores. Neither age, WASI-II FS IQ score, nor ADOS-2 Overall Total SA and RRB scores were significantly correlated with any of the four SRS-2 Subscale Scores shown in Table 1. None of the four SRS-2 Social Communication and Interaction T Scores were significantly different from each other (all *p* > 0.156). As is often expected with psychological data such as these, there was evidence of non-normality in Table 1 data, justifying the choice of a non-parametric statistical procedure for the major data analyses (i.e., Spearman’s correlation coefficient).

### 3.2. Social Communication and Interaction and Anxiety

As mentioned in Hypothesis 1, the associations between GAD and the four SRS-2 subscales that comprise the social communication and interaction variable were used to define the ‘background’ anxiety state that autistic participants experienced in relation to the four aspects of this variable. Table 2 presents those correlation coefficients, indicating that participants’ ability to understand the social demands they were meeting (Social *Cognition*) plus their ability to formulate a satisfactory social response to those demands (Social *Communication*) were meaningfully associated with their GAD levels.

### 3.3. Association Between SRS-2 Subscale Scores and Brain Activity

**Figure 1:**
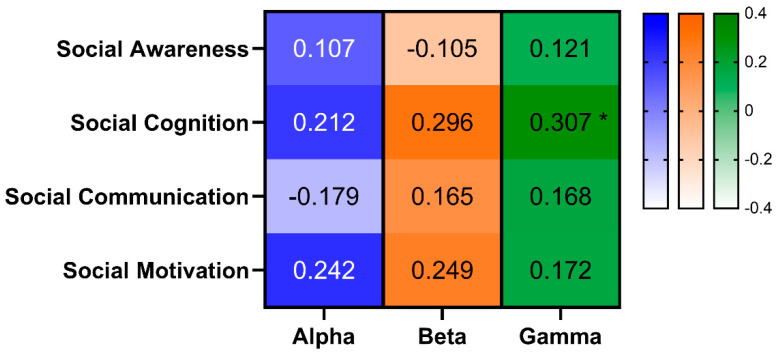
Heat maps of Spearman correlation coefficients between alpha, beta, and gamma power and four SRS-2 subscale T scores in 41 autistic boys. * *ρ* > 0.3.

Hypothesis 2 was focused upon the presence of associations between SRS-2 subscale scores and participants’ alpha, beta, and gamma wave activity collected during the second stage of the study. Figure 1 shows those heat maps of Spearman’s correlation coefficients between each of the four SRS-2 subscale T scores and the total alpha, beta, and gamma activity as calculated by *eLORETA* [23]. Only gamma wave activity reached a medium level of association (i.e., *ρ* ≥ 0.3) [12] with Social *Cognition*, although there were several non-trivial (i.e., *ρ* > 0.20) correlation coefficients between alpha and beta wave activity and Social *Cognition*, and between alpha and beta wave activity and Social *Motivation*.

### 3.4. Hypothesis Testing

Therefore, from these findings, Hypothesis 1 (a significant direct correlation between participants’ SRS-2 subscales and their GAD) was partially accepted as an indicator of participants’ ‘normal’ or ‘background’ state regarding their ability to understand the social communication demands being made on them and their ability to formulate the kinds of social responses required of them. This indicates that poorer social cognition and communication was related to higher anxiety levels in the autistic males in this study.

Hypothesis 2 was also partially accepted in that understanding the social demands made upon them was meaningfully associated in these males with gamma wave levels during a condition of relatively low social demand.

### 3.5. Location of Brain Region Relevant to Social Cognition and Gamma Wave Activity

**Figure 2:**
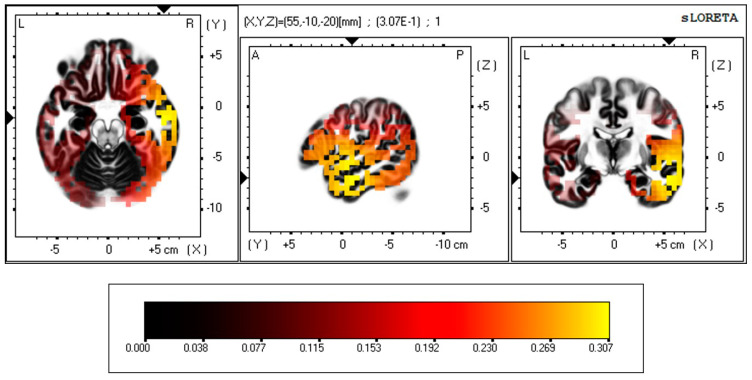
*eLORETA*-sourced regression analysis of changes in gamma band power predicted using SRS-2 Social Cognition score.

Additional to these hypothesis-testing outcomes regarding the strength of associations between SRS-2 Social *Cognition*, GAD, and gamma wave activity reported in Table 2 and Figure 1, regression analyses results from *eLORETA* enabled location of the brain regions where the meaningful association between gamma activity and SRS-2 Social *Cognition* occurred during the low social demand condition. The results of that analysis of the whole brain are presented in Figure 2. The most powerful association between gamma activity and SRS-2 Social *Cognition* occurred in Brodmann Area 21, the middle temporal gyrus, in the temporal lobe. The link between the known functions of this brain’s region and social communication and interaction (particularly Social *Cognition*), and the possible relevance of anxiety to those function, is discussed below.

## 4. Discussion

### 4.1. Major Findings

The key result from this study is the hypervigilance of the adolescent male autistic brain to potentially threatening social interaction demands even when they are not obviously present. It has been well established that difficulties in Social *Cognition* (using Constantino and Gruber’s [2] definition shown in the opening paragraph of the present study) are associated with anxiety when each of those two constructs are measured abstractly via standardized scales under psychological assessment conditions. Those scales are based upon observations of the autistic individual (i.e., the SRS-2) plus the psychophysiological construct of Generalised Anxiety Disorder (GAD) and its diagnostic criteria (i.e., the CASI-4-GAD). The latter are related to the hypothalamus–pituitary–adrenal axis, and they contain fast (adrenaline) and slower (cortisol) hormonal outcomes that prepare the organism for immediate and prolonged ‘anxiety’ responses. It is clear from the previous data regarding this social interaction–GAD connection that social interaction demands can be threatening for autistic persons. Not so well-established is the finding reported here for the parallel anxiety-related neurophysiological response of gamma waves that is designed to bring maximum cognitive effort to the problem of responding appropriately to social demand cues.

### 4.2. Gamma Waves and Anxiety

Gamma waves are generally accepted to occur within the 30–80 Hz range, but they may also be recorded as high as 125 Hz [24], are often studied along with fast (100–400 Hz) and ultra-fast (400–800 Hz) waves, and are thought to facilitate neural communication from the external world to the brain [25]. That neural communication is enhanced by the gamma wave’s linkage of cell assemblies that process related information, such as perception, attentional selection, and memory, enabling them to fire together, so that potentially interfering information is separated in time [26]. As such, gamma waves initiate conditions of maximum concentration and cognitive effort, such as the processing of highly complex information [9], which is perhaps also reflective of the individual’s lack of self-confidence in managing the task and their need to bring maximum resources to it.

Based on the current findings, gamma band power may be considered as an index of high mental workload and cognitive arousal that, when applied to the demands of understanding and responding to social interaction (i.e., Social *Cognition*), represents higher levels of cognitive effort than alpha or beta activity. The need to bring maximum cognitive effort to the task of responding appropriately to social demand cues is emphasized by the fact that those social demand cues represent a major potential social interaction failure point where many autistic individuals are not able to marshal the necessary neurological resources to the task of understanding and responding effectively to their social environment [27]. As such, Social Cognition is threatening and (as indicated by the standardized test data collected here) anxiety provoking. It is entirely expected that the organism might use one of the most powerful forms of neural communication to address the social communication problem underlying this anxiety. Hence, gamma, although not an anxiety response per se (in the same way as the HPA axis), may be conceptualized as a neurophysiological response aimed at reducing anxiety by way of focusing superior neurocognitive facilities upon the problem underlying this anxiety.

### 4.3. Brodmann Area 21 and Social Cognition

Defined by Fernandez et al. [28], ‘social cognition’ consists of perceiving and processing social cues, integrating those cues with internal physiological status, and responding to a particular social demand. The first two stages in this process have received research focus in an effort to understand the neurological phenomena that underly them [29] particularly the effect of specific brain wave activity in particular brain regions [30]. However, those findings were based upon a meta-analysis of 50 neuroimaging studies of social cognition in children and adults with vs. without ASD. While informative, they reflect a good deal of variability across samples and refer to large areas of the brain. Other studies have postulated the presence of ‘social cognitive networks’ based upon the ‘social brain’ (medial prefrontal cortex, orbitofrontal cortex, anterior cingulate cortex, temporoparietal junction, inferior frontal gyrus, and superior temporal sulcus) [31], but those networks were not specific to autism. The current study’s findings have extended that research by identifying the association between gamma wave activity and the social demands as mostly occurring in the specific brain region Brodmann Area 21, the middle temporal gyrus, in the temporal lobe.

The temporal lobe is involved in memory and perception [32], although with a variability in function that has been attributed to single neurons [33], which is suggestive of the capacity to manage many tasks involved in how organisms perceive the external world. Connectivity between the middle temporal gyrus and the hippocampus has been shown to be a key factor in forming new associations between hitherto non-connected entities or concepts [34], and intra-connectivity within the middle temporal gyrus has been linked with social cognition and language [35]. Brodmann Area 21 is approximately in the same region as the middle temporal gyrus, and it is involved with higher-order visual associations as well as auditory processing and language [36]. Taken together, these functions argue for the relevance of gamma activity as a process of focusing relevant cell groups to bring maximum cognitive power to the process of understanding and responding to threatening social cues.

### 4.4. Implications for Assessment and Treatment of Mental Health in Autism

**Figure 3:**
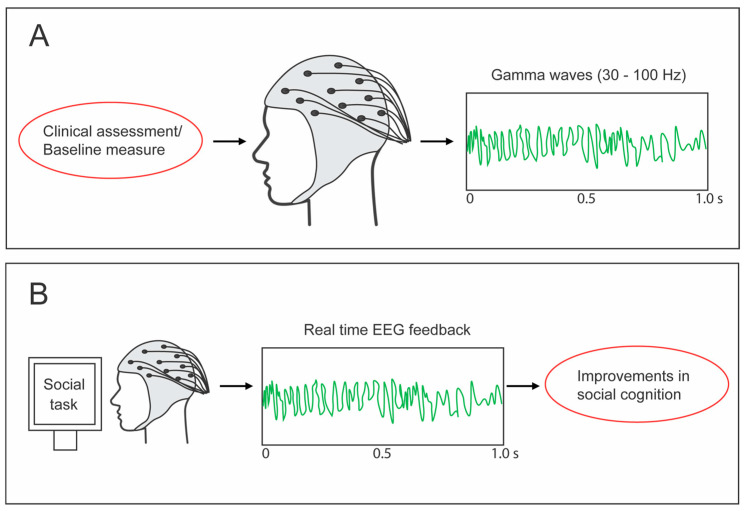
(**A**) Initial assessment of gamma activity, (**B**) EEG gamma feedback training to improve social cognition.

These findings suggest that the collection of gamma data during initial assessment and diagnosis of ASD, and also during times of high, low, effective, and ineffective social interactions, might provide an additional insight into the autistic youth’s need and ability to resolve challenges arising from social communication and interaction difficulties. Similar to most anxiety responses (e.g., elevated HPA-axis activity), elevated gamma can be conceptualized as a factor designed to increase the individual’s opportunities for success, but instead of ‘fight or flight’, gamma may work through enhancement of the individual’s ability to problem-solve. As a baseline indication of how well prepared autistic youth are to face the complex challenges of social communication and interaction, resting gamma wave power, particularly in brain regions such as BA 21, could be a valuable item of assessment profile data. Figure 3A outlines this process.

Concurrently, because there is reduced spontaneous gamma activity in autistic vs. non-autistic youth in the frontal, temporal, and right-lateral regions, as well as during sessions designed to evoke gamma [37], the incorporation of gamma training regimes for autistic youth could represent an avenue towards increasing gamma activity when and where it may play an expeditious role in helping the autistic individual understand social cues [38]. Various forms of transcranial stimulation have been demonstrated to induce elevations in autistic participants’ gamma activity [39], and neurofeedback has also been shown to be successful in this regard [40] (see Figure 3B). There may be some advantage in focusing that training upon BA 21, as shown in Figure 3B.

### 4.5. Limitations

Although acceptably large in this field of research, replication with a larger sample of autistic males would support the generalisation of these results. Autistic boys were selected because of the imbalance in ASD prevalence across males and females, but an extension of this study to include a comparable sample of autistic girls is necessary to understand any possible sex effects upon these findings. The SRS-2 and CASI-4-GAD are well known in the field of autism research as being valid and reliable, and self-reports of GAD by young autistic males have been previously reported to have higher agreement with a biological indicator of stress and anxiety than parent reports [41]. However, further information on the two sets of variables (GAD, social communication and interaction) from other sources would add validity to these procedures. The two studies conducted here did not include comparison with a social interaction per se, and the collection of more ‘real world’ social interaction data plus EEG recording is the next step in this research. Similarly, the collection of data at a single point in time does not address questions related to variability in social anxiety. Larger samples would enable age subgroup comparisons, possibly testing the effects of puberty. Notwithstanding these limitations, these results provide an initial insight into how gamma waves may operate within the anxiety framework that is often present when autistic youth seek to socially interact.

### 4.6. Conclusion: Social Cognition, Gamma Activity, Anxiety, and Autistic Mental Health

Anxiety is often comorbid in autistic children [42], and it interferes with the daily functioning of these children [43]. As well as the traditional anxiety responses that produce faster or slower physiological reactions (e.g., HPA-axis-induced adrenalin and cortisol), the perception of imminent threat that involves problem solving (rather than flight or fight) can initiate a different fear-mediating response—enhanced gamma wave activity. Particularly relevant to the brain regions shown in Figure 2 for the direct marshalling of strong cognitive resources to understand the nature of fearful social cues and then develop effective responses to those cues, gamma waves represent an important aspect of the autistic youth’s response to their ASD-intrinsic difficulty in social communication and interaction. As such, the recognition of this aspect of the neurophysiological response systems that the young autistic person possesses is overdue not only in its specific role in reducing the threat involved in social interaction but also as a relevant factor in the overall mental health of young autistic people. As well as the specific suggestions for future research described in Section 4.4 and Section 4.5, these results also support focused examination of the neurological pathways that are used to process emotional information in a range of psychiatric disorders in order to identify any commonalities or peculiarities according to specific disorder, which may then hold implications for treatment choices. Similarly, further investigation of the ways that autistic youth use gamma wave activity to deal with various subtypes of anxiety (separation anxiety, social anxiety, panic disorder, etc.) as they encounter other major sources of anxiety in their lives has the potential to suggest possible treatment models.

## Figures and Tables

**Table 1:** Mean (SD, range) data from 41 autistic boys for age, WASI-II Full Scale IQ, and ADOS-2 Overall Total SA + RRB score, CASI-4-GAD, and SRS-2 Subscale T Scores for Social Communication and Interaction.

	Age (yr)	WASI-II Full Scale IQ	ADOS-2 Overall Total SA + RRB Score	CASI-4-GAD
Mean	10.76	102.10	9.49	9.14
SD	3.13	14.46	0.870	4.62
Maximum	6	77	7	0
Minimum	17	130	10	20
SRS-2	Social Awareness T Score	Social Cognition T Score	Social Communication T Score	Social Motivation T Score
Mean	71.05	74.00	74.85	67.61
SD	12.08	10.11	11.27	12.87
Maximum	38	40	46	44
Minimum	90	90	90	90

**Table 2:** Spearman correlation coefficients between SRS-2 subscale T scores and CASI-4-GAD score in 41 autistic boys.

SRS-2 Subscale	CASI-4-GAD
Social awareness	0.128
Social Cognition	0.334 *
Social Communication	0.347 *
Social Motivation	0.276

* *ρ* > 0.3, *p* < 0.05.

## Data Availability

The raw data supporting the conclusions of this article will be made available by the authors upon request.
